# Visitors at Surgical Operations

**Published:** 1912-07-27

**Authors:** 


### The Hospital

The Workers' Newspaper of Administrative Medicine and Institutional Life, Administration, National Insurance and Health.

No. 1360, Vol. LII. SATURDAY, JULY 27, 1912. PRICE ONE PENNY.

### VISITORS AT SURGICAL OPERATIONS.

A short paragraph in the issue of The Hospital for July 20 (page 401), which details a proposal adopted at a recent meeting of the Board of Management of King Edward VII.'s Hospital, Cardiff, raises issues of considerable moment to those who have control of the operating theatres of the voluntary hospitals. It will be remembered that Major-General Lee moved a resolution on this topic which was amended to the effect that the only visitors to be allowed at operations should be qualified medical men, medical students, and trained nurses. It would appear from General Lee's arguments in support of the regulation that applications for educational facilities (by admission to the operating theatres) are frequently received from members of first aid and other societies; and it is common knowledge that Cardiff is not the only town where this thirst for knowledge exists and asserts itself.

There is a good deal to be said both *pro* and *con* concerning the principle which has been at stake in Cardiff. To take first the case for allowing certain laymen (and laywomen) in certain circumstances to be present at surgical operations. The last few years have witnessed an enormous extension of first aid and ambulance activity, partly owing to the formation of the Voluntary Aid Detachments of the Territorial Force. It can hardly be contended that admission to a hospital ward or theatre is necessary to enable anyone to comprehend the rudiments of first aid to the injured as taught in the elementary classes of the St. John's Ambulance Association or of the Red Cross Society. But the activities of these bodies do not now stop at elementary first aid to the injured. They include home nursing and similar subjects as well, and they also provide advanced instruction for more extensive curricula. Thus it comes about that an opportunity of seeing aseptic and antiseptic technique in full working order is undoubtedly helpful to many non-medical persons by enabling them to assimilate the instruction that is being offered them. Further, such principles as those of hæmostasis and of the treatment of surgical shock are constantly being exemplified in any operating theatre; and these are admittedly of high value to members of voluntary aid detachments or anyone else who may some day have to succour the wounded on a battle-field.

We may reasonably, therefore, concede that admission to an operating theatre may, in certain circumstances, be of benefit to non-medical persons. Obviously, such a privilege should be hedged about with stringent conditions to prevent its abuse. The right should be reserved to exclude such visitors for the whole or part of any operation at the discretion of the operator. The visit should never be allowed save in the personal charge of a qualified practitioner able and willing to make himself responsible for the conduct and character of his protégés. It may also be argued that when spinal anæsthesia is being employed there is in any case one non-medical conscious visitor to an operating theatre, namely, the patient himself; and it has never been contended that such a visitor is objectionable.

On the other hand, it is easy to point out excellent reasons why the precedent set at Cardiff may well be followed elsewhere. In the first place, it is not for hospital and surgical authorities to show cause against the intrusion of lay visitors at operations, but rather for the latter to prove that any need for such admission outweighs its disadvantages. The onus of proof, in fact, lies with those who countenance the innovation. Failing such evidence, the public, or selected members of it, can have no grievance against rules such as those passed at the King Edward VII.'s Hospital. The next point is that all visitors at operations are of necessity a hindrance to the business in hand. Even qualified practitioners, and *a fortiori* medical students, often get in the way of the nurses, surgeons, anæsthetists, or other active participators in operations. For good and sufficient reasons, visitors of this class are often admitted almost as a matter of course, but in actual strictness they are present by permission of the operator, expressed or implied. Such professional visitors can and do minimise the inconvenience of their presence, because they know what to do and what not to do. They recognise the importance of silence and stillness, and of doing nothing to distract anyone taking part or assisting in the operation.

Further, they take good care to do nothing which shall imperil the Listerian principles on whose observance the success of the operation depends; and it is fatally easy for the untrained and the ignorant to vitiate in a moment these precautions, and so to jeopardise the health and even the life of the patient. Furthermore, it is not unknown for lay visitors to operating theatres to require professional services on account of faintness or other symptoms caused by the unaccustomed sights and smells about them.

Furthermore, the lay visitor at an operation gains really nothing by watching the manipulations of the surgeon, whose object and methods he cannot possibly appreciate. There is, in consequence, always the danger that he may constitute himself a critic of that which he does not understand, and so cause unnecessary friction in a score of ways. As for antiseptic technique, if he must see it in practical application, the surgical wards, and especially the casualty rooms, afford a much more useful illustration of it (from the first aid standpoint) than do the operating theatres.

